# Copper–PLLA-Based Biopolymer Wrinkle Structures for Enhanced Antibacterial Activity

**DOI:** 10.3390/polym17162173

**Published:** 2025-08-08

**Authors:** Petr Slepička, Iva Labíková, Bára Frýdlová, Aneta Pagáčová, Nikola Slepičková Kasálková, Petr Sajdl, Václav Švorčík

**Affiliations:** 1 Department of Solid State Engineering, University of Chemistry and Technology Prague, 166 28 Prague, Czech Republic; iva.michaljanicova@gmail.com (I.L.); bara.frydlova@vscht.cz (B.F.); nikola.kasalkova@vscht.cz (N.S.K.); vaclav.svorcik@vscht.cz (V.Š.); 2 Department of Biochemistry and Microbiology, University of Chemistry and Technology Prague, 166 28 Prague, Czech Republic; pagacova@vscht.cz; 3 Department of Power Engineering, University of Chemistry and Technology Prague, 166 28 Prague, Czech Republic; petr.sajdl@vscht.cz

**Keywords:** biopolymer, poly-L-lactic acid (PLLA), wrinkle structure, copper composites, surface morphology, antibacterial properties

## Abstract

The increasing prevalence of antibiotic-resistant bacteria has intensified the need for innovative antibacterial surfaces, particularly in biomedical applications. Traditional approaches often rely on chemical agents alone, which may lead to diminishing efficacy over time. To address this, we investigated the development of a novel antibacterial surface by combining the inherent antimicrobial properties of copper with an engineered surface topography on a biopolymer matrix. A copper–poly-L-lactic acid (Cu-PLLA) composite system was fabricated using sputtering deposition followed by controlled thermal treatment to induce wrinkle-like micro- and nanostructures on the surface. The surface morphology was characterized using scanning electron microscopy (SEM) and atomic force microscopy (AFM), confirming the formation of hierarchical wrinkle patterns. The chemical composition and distribution of copper were analyzed via energy-dispersive X-ray spectroscopy (EDS). Antibacterial performance was assessed against both Gram-negative *Escherichia coli* and Gram-positive *Staphylococcus aureus* using standard colony count reduction assays. The Cu-PLLA wrinkled surfaces demonstrated significantly enhanced bactericidal activity compared with flat PLLA and copper-free controls, a finding attributed to a synergistic effect of mechanical membrane disruption and copper-mediated chemical toxicity. These findings suggest that biopolymer–metal hybrid surfaces with engineered topography offer a promising strategy for developing next-generation antibacterial materials suitable for biomedical and clinical use.

## 1. Introduction

The development of advanced biomaterials with inherent antibacterial functionality has become a necessity in modern medicine, particularly due to the alarming rise in antibiotic-resistant bacterial strains. Implantable medical devices, wound dressings, and tissue-engineering scaffolds are often subject to bacterial contamination, leading to infection, delayed healing, or implant failure. Consequently, multifunctional surfaces that prevent bacterial colonization while supporting mammalian cell compatibility are highly desirable [1,2]. Among the strategies developed to achieve antibacterial activity, surface topography engineering and metal incorporation have emerged as two of the most effective and versatile approaches. Recent studies have shown that specific micro- and nanoscale surface patterns, particularly those mimicking natural antimicrobial surfaces (such as insect wings or shark skin), can significantly inhibit bacterial adhesion and growth through mechanical disruption of bacterial membranes [3,4]. Among biodegradable polymers, poly-L-lactic acid (PLLA) has been widely studied due to its biocompatibility, biodegradability, and FDA approval for various biomedical uses [5,6]. However, its inherently smooth and chemically inert surface often lacks the properties necessary to deter bacterial colonization; recent advancements have sought to enhance PLLA’s functionality by introducing surface modifications such as plasma treatments, and specific techniques such as improved phase separation create patterns [7,8,9,10] that can modulate both bacterial and eukaryotic cell responses.

Wrinkling instability is a bottom-up, self-organizing approach in which uneven expansion of surfaces with different mechanical properties leads to wrinkle formation [11]. This phenomenon is observed when the residual stress exceeds a critical value, triggered by various factors such as thermal effects [12,13], solvent swelling [14], mechanical stretching, or contraction [15,16]. For instance, heating a thin polymer–metal bilayer film induces wrinkling due to mismatched thermal expansion coefficients between the layers [13,17]. Studies have demonstrated that heating below the glass transition temperature (Tg) of polymers does not alter the surface morphology, while temperatures above Tg result in the desired wrinkle formation [18]. Similar studies have investigated systems with various combinations of polymers and metals, including gold [19] and platinum [20].

The antimicrobial properties of copper were first documented in Egyptian medical texts from 2200 to 2600 BC, where it was used for water sterilization and wound treatment [21]. Today, copper nanoparticles are recognized for their potent antimicrobial properties, finding significant applications in antibacterial surfaces and medicine. These nanoparticles exhibit strong activity against bacteria, viruses, and fungi, making them effective against pathogens, including drug-resistant microorganisms such as methicillin-resistant *Staphylococcus aureus* (MRSA) [22,23,24,25]. In addition, recent studies show that copper effectively inactivates the COVID-19 virus [26]. In the field of electronics, copper is more cost effective than precious metals and offers excellent electrical conductivity, making it an ideal material for various applications [27,28]. This dual applicability underscores the versatility in both medical and electronic fields. The biopolymer wrinkles can be finely tuned in wavelength and amplitude [29] to physically interfere with bacterial cell membranes while maintaining a conducive environment for mammalian cell adhesion and proliferation. Notably, wrinkled surfaces have demonstrated reduced surface area available for bacterial adhesion and increased local curvature, which enhances antibacterial effects via mechanical stress on bacterial membranes [30]. Copper-based nanocomposites have shown efficacy against both Gram-positive and Gram-negative bacteria, and their integration into polymeric biomaterials is a promising strategy for infection-resistant surfaces [31]. Recent research suggests that such hybrid materials can outperform their individual components, offering enhanced antimicrobial resistance, mechanical stability, and long-term biofunctionality [32]. Moreover, studies have confirmed that controlled copper release from polymer matrices can be tailored to maximize bactericidal action while minimizing cytotoxicity to host tissues [33,34].

Several studies aimed at modifying/enhancing the antibacterial properties of PLA-based composites have been published. Electrospun PLA/ZnO composite films have shown enhanced antibacterial properties and been applied in the food industry [35]. The antibacterial, crystallization, and mechanical properties of PLA were modified with the addition of chitosan-based materials [36]. A combination of alkaline treatment and PLA/f-CNT composite coating influenced the corrosion, biocompatibility, and antibacterial activity of Mg alloy [37]. The biomass carbon dots and the composite nanofiber membranes of biomass carbon dots and PLA, along with their photocatalytic degradation and bio-antimicrobial properties, were studied in [38]. Lee at al. prepared an antibacterial PLA/Mg composite with enhanced mechanical and biological performance for biodegradable orthopedic implants [39]. Silver nanoparticles were used in aerogels to improve their antibacterial properties [40]. Romero et al. prepared antibacterial and biocompatible polymeric composites with copper zeolite filler and copper oxide nanoparticles [41]. General strategies for the preparation of polylactic acid composites with UV resistance and antibacterial properties were introduced in [42].

The Cu-PLLA wrinkle surfaces developed in this study have strong potential for applications in a variety of fields in which bacterial contamination poses a critical risk. Notably, these surfaces could be implemented in biomedical devices such as catheters, surgical tools, wound dressings, and implantable components [43] in which infection prevention is paramount and in which silver is also tested [44]. Beyond the medical sector, applications in food packaging, water filtration membranes, and high-contact surfaces in public or industrial settings (e.g., door handles, railings, or touchscreens) are also promising. The integration of copper as an antimicrobial agent, combined with the physical bactericidal effects of nanoscale topographies, offers a dual-action mechanism that may reduce reliance on antibiotics and chemical disinfectants. From a cost perspective, the fabrication method employed—thermal wrinkling combined with sputtering—is relatively straightforward and compatible with existing industrial processes [45]. Compared with traditional antibacterial coatings, such as those based solely on silver nanoparticles or complex polymer–antibiotic conjugates, the Cu-PLLA approach is more cost effective due to the lower material costs of copper and the potential for scalable patterning without the need for lithography or cleanroom conditions. Additionally, the use of biodegradable PLLA as the base polymer aligns with sustainable material strategies and may lower end-of-life disposal costs. Overall, this system demonstrates a favorable balance between performance, material cost, and manufacturability, supporting its feasibility for real-world adoption across both high-tech and commodity product sectors.

Even details of the antibacterial properties of copper in different materials, such as acrylonitrile styrene acrylate [31], and other polymeric nanocomposites, the combination of copper and poly-L-lactic acid enhanced with surface wrinkling, have not been published to date, to the best of our knowledge. Despite growing interest in this area, few studies have systematically explored copper-integrated PLLA surfaces with wrinkled microarchitecture for antibacterial applications. Understanding the interplay between surface morphology, chemical composition, and biological response remains crucial. The present work addresses this gap by developing and characterizing wrinkled Cu–PLLA surfaces and evaluating their antibacterial efficacy against both *E. coli* and *S. aureus*, which serve as representative models for Gram-negative and Gram-positive bacterial strains, respectively. The use of wrinkle engineering combined with bimetallic or monometallic copper deposition and solvent-based shaping techniques offers a novel platform for the next generation of infection-resistant biomaterials.

## 2. Materials and Methods

### 2.1. Materials

Biopolymer poly-L-lactic acid (PLLA) (density 1.25 g·cm^−3^, glass transition temperature T_g_ = 60 °C, crystallinity 60–70%, thickness 50 μm; Goodfellow Ltd., Huntingdon, UK, elongation at break 6%, tensile modulus 3.5 GPa) was used in the experiments. The polymer foil was modified via copper sputtering using Quorum 300T ES system (Quorum Technologies, Laughton, UK). The typical sputtering conditions were room temperature, sputter time 50–400 s, argon gas with 99.997% purity at a pressure of 1 Pa, and sputtering current of 20–60 mA.

### 2.2. Analytical Methods

#### 2.2.1. Atomic Force Microscopy

The surface morphology and roughness of the pristine and treated films were examined with atomic force microscopy (AFM) using a Dimension ICON (Bruker Corp., Billerica, MA, USA). The samples were analyzed in Scan-Assyst^®^ mode using a nitride lever SCANASYST-AIR (Bruker Corp., Billerica, MA, USA) with a Si tip (spring constant of 0.4 N·m^−1^). NanoScope Analysis 1.80 software was applied for data processing. The surface roughness (R_a_) represents the arithmetic mean of the absolute values of the height deviations measured from the central plane; RMS represents the root mean square variation.

#### 2.2.2. Scanning Electron Microscopy

The morphology of the sample surfaces was also characterized with a scanning electron microscope FIB-SEM LYRA3 GMU (Tescan, Brno, Czech Republic). The acceleration voltage was set to 10 kV. The elemental composition was measured using energy-dispersive X-ray spectroscopy (EDS, analyzer X-ManN, 20 mm^2^ SDD detector, Oxford Instruments, Oxford, UK), while the accelerating voltage for SEM-EDS analysis was set to 10 kV.

#### 2.2.3. X-Ray Photoelectron Spectrometry

The elemental composition on the material surface was analyzed with X-ray photoelectron spectroscopy (XPS) using a spectrometer ESCAProbeP (Omicron Nanotechnology Ltd., Taunusstein, Germany). As a source, a monochromatic X-ray at an energy of 1486.7 eV was used. The atomic concentrations of the elements were determined from the individual peak areas using CasaXPS software 2.3.17PR 1.1.

#### 2.2.4. Wettability

The wettability of the studied samples was determined by measuring the contact angles (CA, θ) using a goniometer (Advex Instruments, Brno, Czech Republic) connected to the SEE System 7.6 program. Analysis of CA was performed at room temperature with 8 µL drops of distilled water using a Transferpette^®^ automatic pipette (Brand, Wertheim, Germany) at 6 different positions of 3 samples in parallel and perpendicular directions. Subsequently, the drops were photographed and evaluated at 3 marked points. Estimation of surface energy was also based on the measurement with the SEE System. Two liquids (water and glycerol) were used for the measurements. On the base of the Owens–Wendt method, the values of contact angles were evaluated. The measurement was carried out at room temperature. Values of surface energy were consequently evaluated with the Origin 8.0 software.

### 2.3. Antibacterial Testing

The antibacterial activity of the samples was determined using the so-called drip test performed with *Escherichia coli* representing Gram-negative bacteria and *Staphylococcus aureus* representing Gram-positive bacteria. From agar plates of *E. coli* and *S. aureus* strains, one colony each was transferred into 20 and 5 mL of liquid Luria–Bertani (LB) medium, respectively. The inocula were then incubated overnight in an orbital shaker at 37 °C. The following day, the bacterial suspensions were diluted with phosphate-buffered saline (PBS) to a concentration of approximately 1.5 × 10^4^ bacteria/mL for *E. coli* and 1.3 × 10^4^ bacteria/mL for *S. aureus*. The test samples were dipped in triplicates into 2 mL of diluted bacterial suspension and statically incubated at laboratory temperature. A bacterial suspension without added sample was prepared and incubated in the same manner to control bacterial growth, and a PBS solution alone was used to control contamination. After 2 and 24 h, the samples were mixed, and 5 drops of 25 µL each were dropped onto PCA plates (plate count agar). These plates were then cultured at 28 °C until colonies were visible and suitable for counting in the samples to control bacterial growth. The number of colony-forming units (CFUs) was then determined and compared with the CFU count in the bacterial growth control. The experiment was conducted under sterile conditions.

## 3. Results

### 3.1. Atomic Force Microscopy

**Figure 1:**
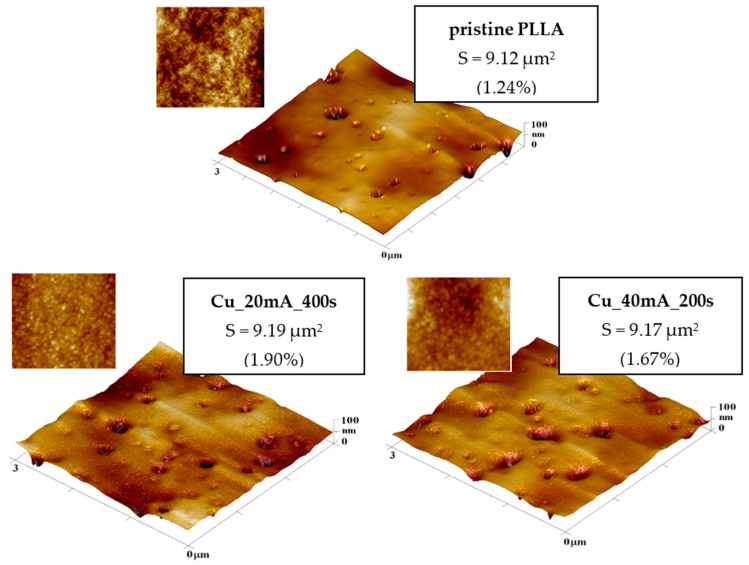
Surface morphology of pristine PLLA, sputtered PLLA with Cu: left, sputtering current 20 mA and sputtering time 400 s; right, sputtering current 40 mA and sputtering time 200 s.

The surface morphology of sputtered poly-L-lactic acid foil is shown in Figure 1. Two different Cu thicknesses on PLLA foil are presented; for the sake of clarity, the detailed morphology is of the 3 × 3 μm^2^ thickness only. As is evident from this figure, no significant change in surface morphology is associated with the increasing thickness (sputtering time of Cu). The inset of the figure represents the square of 300 × 300 nm^2^, where the globular pattern formed on the surface is evident.

It is evident from the values of surface average roughness and effective surface area that the copper sputtering does not change the surface morphology significantly. It is also important to say that we did not introduce the surface morphology of pristine PLLA, since no significant changes compared with sputtered surfaces were observed, except that the detailed surface morphology of pristine PLLA lacks a globular pattern.

Although the sputtered globular structure is present, a much “smoother” surface and smaller globules compared with those observed, e.g., for gold [46] were detected. The Cu-sputtered PLLA foils with different thicknesses of Cu were subsequently heat-treated at the PLLA glass transition temperature for one hour. Our previously acquired results confirmed that, with increasing thickness of noble metal, a wrinkle-like pattern is induced on the surface of the noble metal–PLLA system. This phenomenon was for the first time confirmed for Cu on PLLA. What is connected with this phenomenon? During the heat treatment, when it is close to the glass transition temperature of PLLA, a bilayer of copper-enhanced PLLA is formed on the surface of the bulk PLLA foil. This bilayer has some specific properties, such as increased thermal conductivity. Therefore, during this procedure, and after the removal of the Cu-enhanced sample from the oven during the cooling procedure (a rather rapid phenomenon considering the system and the material—within tens of ms), wrinkling instability occurs. Compared with the bulk foil, the enhanced thermal treatment induces the wrinkle pattern, which is directly connected to the amount of copper and, therefore, the thickness of the copper layer on the PLLA foil.

Wrinkling occurs in a film supported by a soft substrate due to the deformation mismatch once the compressive stress exceeds a threshold value. Various methods associated with thermal deposition, plasma, ultraviolet/ozone treatment, mechanical stretching, and solvent swelling [18,19,47,48] have been developed to induce ordered wrinkles; in our case, thermal treatment was applied. On the basis of the principle of minimizing the total potential energy, when the cooling procedure is applied to the surface [47,48], the polymer/metal bilayer differs from the remaining bulk due to better heat conductivity, and a wrinkle pattern is formed. The wrinkle wavelength depends on film thickness and the elastic moduli of films and substrates, while the wrinkle amplitude is related to not only film thickness and the elastic moduli of films/substrates but also compressive strain, and the biaxial or uniaxial film orientation may also play an important role [19,20].

**Figure 2:**
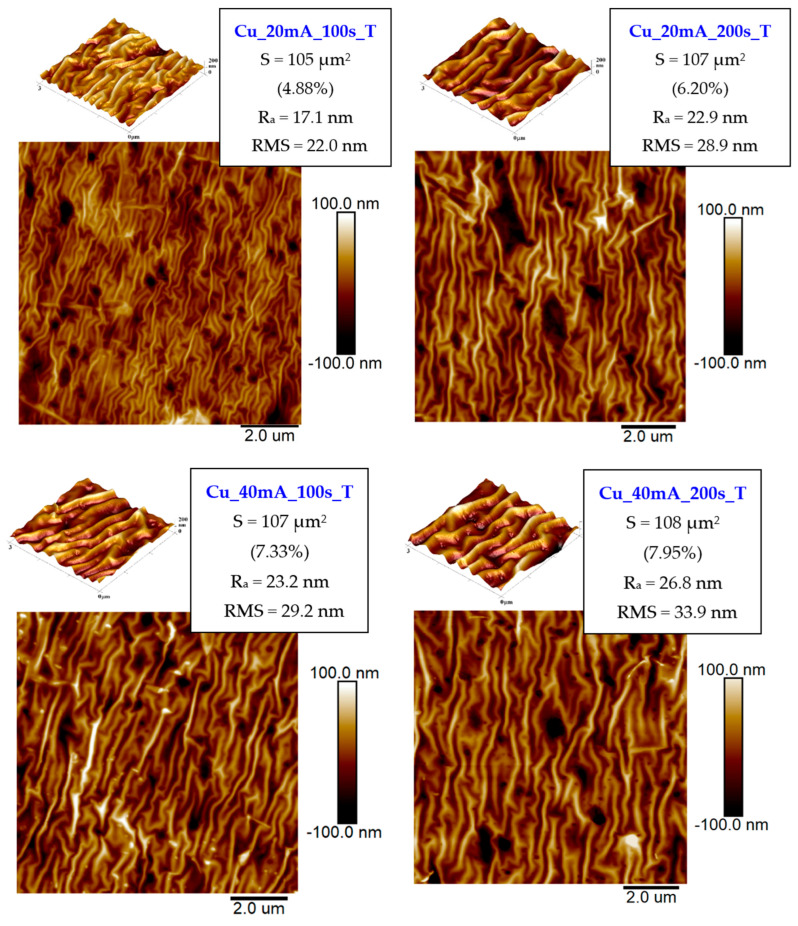
Surface morphology of sputtered PLLA with Cu and subsequently heat-treated (1 h, 60 °C): sputtering currents of 20 mA and 40 mA (100 s and 200 s). The effective surface area (S), average surface roughness (R_a_), and root mean square roughness (RMS) are shown.

As shown in Figure 2, the wrinkling appears when the thermal treatment is applied to the PLLA, in accordance with the effect described in the previous paragraph. The amplitude of the wrinkle pattern and the density of the wrinkles are strongly dependent on the thickness of the metal layer (and, therefore, the sputtering current and time). With the increasing thickness of the Cu layer, the wrinkle pattern is more pronounced after the heat treatment procedure. With an increasing amount of metal present on the PLLA, the surface roughness and the surface effective area also increase, both for the increasing sputtering time and the sputtering current, as is evident from Figure 2. The wrinkling instability leading to the inducement of the surface wrinkle pattern may also significantly affect the antibacterial properties of prepared surfaces, as described below. Additional AFM scans for a 40 mA current and different sputtering times are introduced in Figure S1.

### 3.2. Scanning Electron Microscopy and Energy Dispersive Analysis

**Figure 3:**
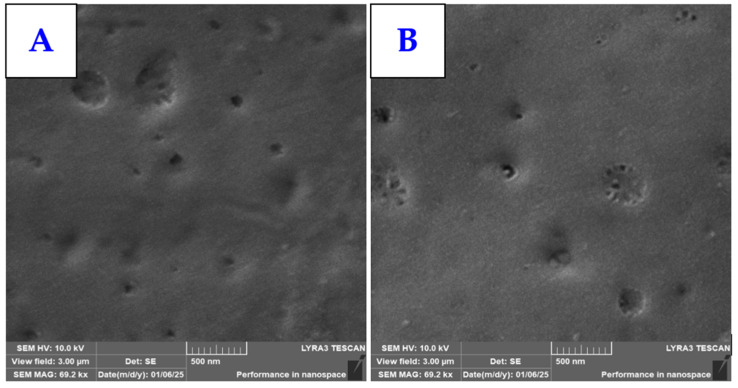
Surface morphology (SEM imagery) of sputtered PLLA with Cu: sputtering current 20 mA and 200 s (**A**); sputtering current 40 mA and 200 s (**B**).

The significant change in surface morphology induced by the combination of Cu sputtering on the PLLA surface and subsequent heat treatment at a temperature close to the glass transition temperature of PLLA biopolymer was also confirmed by scanning electron microscopy analysis. For comparison, we first analyzed the samples with different thicknesses of Cu nanolayers on the PLLA surface (20 mA and 40 mA, 200 s), as shown in Figure 3. As expected, the sputtering of the thin metal nanolayers did not induce any significant changes on the surface of the PLLA biopolymer. The globular pattern structure visible in the AFM analysis results is not clearly visible in the SEM images.

**Figure 4:**
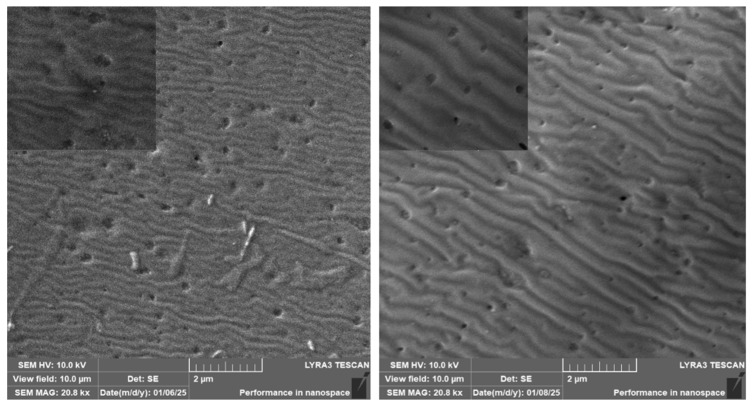
Surface morphology (SEM image) of sputtered PLLA with Cu: left, sputtering current 20 mA and 200 s; right, 40 mA and 200 s, subsequently heat-treated at 60 °C for 1 h. Inset image represents always the 3 micron scan for particular sample.

The surface morphology of the heat-treated PLLA with deposited copper is shown in Figure 4. We selected for the comparison the same sputtering times and different sputtering currents of 20 mA and 40 mA, the “40 mA” sample having approximately doubled the Cu layer on its surface.

Figure 4 clearly indicates that, with an increasing amount of Cu on the surface, the period of the ripple pattern increases after heat treatment. As shown in the figure, the homogeneity of the surface ripple also increased, and in correlation with the AFM images, the period of the pattern increased approximately in the same ratio as the deposited Cu thickness (double the thickness, double the pattern period).

**Figure 5:**
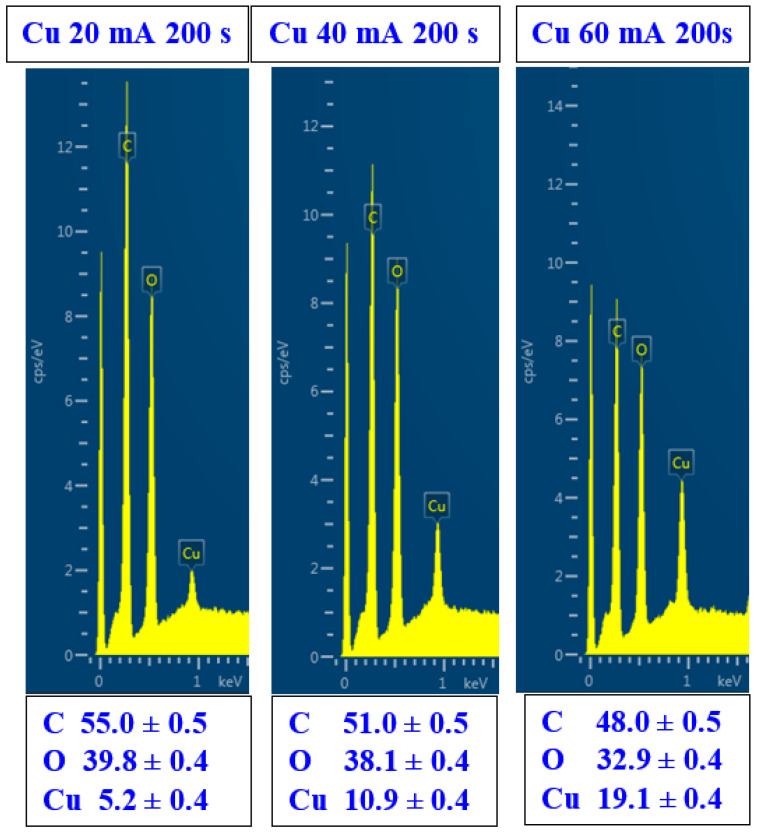
Surface chemistry (EDS spectra) of sputtered PLLA with Cu: sputtering current 20 mA, 40 mA, and 60 mA with sputtering time 200 s.

We also determined the surface chemistry of both the Cu-deposited and subsequently heat-treated PLLA surfaces. As Figure 5 clearly shows, even for a very thin Cu layer, we detected 5.2 wt. % of Cu on the sputtered surface. With an increasing sputtering current, the concentration of Cu increased, in good correlation with the increasing sputtering current. The surface deposited with 40 mA exhibited a Cu concentration of 10.9%, while for 60 mA, we detected a Cu concentration of 19.1%. As is obvious from these data, the oxygen concentration also decreases, which indicates that a non-oxidized layer is formed on the PLLA surface. However, the oxidation of Cu is discussed below in the section devoted to the XPS analysis.

**Figure 6:**
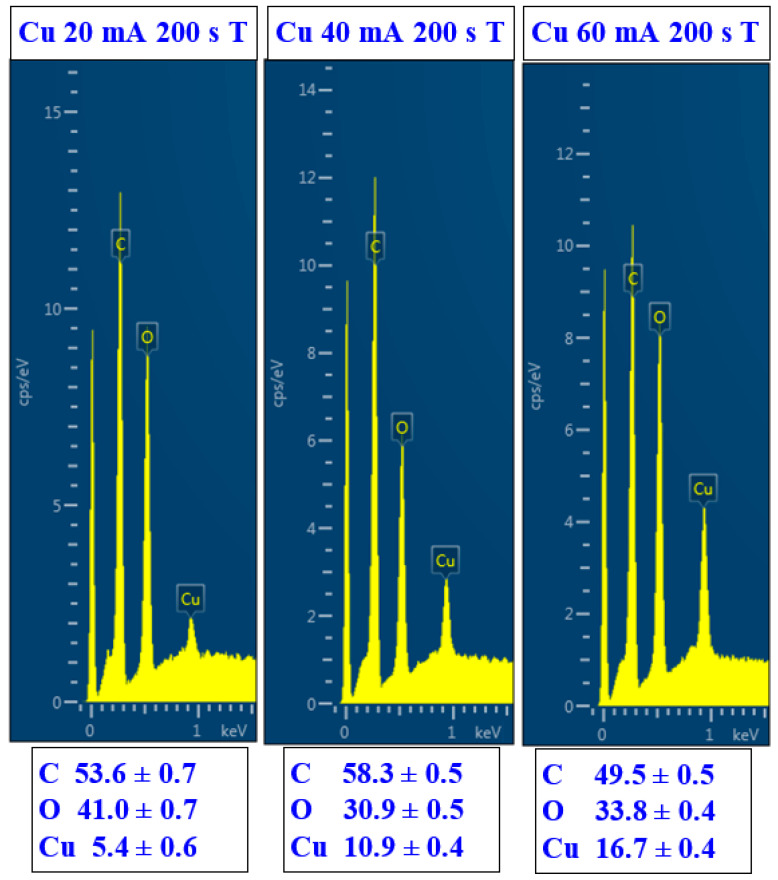
Surface chemistry (EDS spectra) of sputtered PLLA with Cu: sputtering current 20 mA, 40 mA, and 60 mA with sputtering time 200 s. All samples were subsequently heat-treated at 60 °C for 1 h.

The major phenomenon that occurred during the heat treatment of the Cu-sputtered PLLA was the wrinkle pattern formation. This was connected with the formation of a Cu-PLLA composite bilayer, as discussed above. Therefore, we also aimed to study the surface chemistry changes when the ripple formation is detected. The EDS spectra of the same set of samples described in the previous paragraph are presented in Figure 6 for the heat-treated specimens. Although the wrinkle pattern is induced, the amount of Cu in the wrinkled surface layer is not significantly altered; we observed a maximum decrease from 19.1 to 16.7 wt. % for the sample deposited with the highest current 60 mA. We have to note that the ability of EDS analysis to acquire surface elemental concentration is up to several hundreds of nm; therefore, although there was spatial redistribution of Cu from the surface into the ripple composite, no significant differences were expected. As stated above, the only exception was the sample with the largest amount of Cu on the surface (60 mA), where the lateral period of the wrinkles was the highest; in this case, some of the copper was diffused far from the surface, and a lower Cu concentration was observed. A rather different situation can be expected as we analyze the surface layer and surface elemental concentration, which will be described in the next section devoted to the XPS analysis of both deposited and wrinkled surface patterns.

### 3.3. X-Ray Photoelectron Spectroscopy

We aimed to perform an elemental analysis of the surface and to study the oxygen and copper concentration, both for the copper-deposited samples and subsequently heat-treated samples.

**Figure 7:**
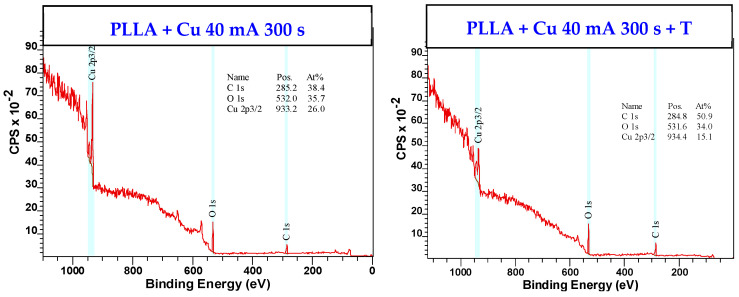
Surface elemental chemistry (XPS spectra) of PLLA sputtered with Cu: sputtering current 40 mA with sputtering time 300 s, and the same sample subsequently heat-treated at 60 °C for 1 h (T).

Since the wrinkle pattern formation is connected with the diffusion into the biopolymer [19,20], we studied the elemental concentration using two techniques, including XPS, which is designed to reveal changes on the surface. As expected, the pristine PLLA surface revealed only the presence of oxygen and carbon. The sputtering of copper significantly increased the copper concentration (17.6 at. % for 50 s deposition, and up to 26.0 at. % for 300 s deposition) (for spectra and data, see Table 1 and Figure 7). As this figure shows, the amount of detected copper is relatively high even for a very short sputtering time, if we compare the results with EDS spectra and concentrations acquired using EDS analysis (Figure 5). Some additional XPS spectra can be found in Figure S2.

Even the absolute concentrations are different, and the Cu concentration acquired with XPS is higher compared with that for EDS analysis; the trend of Cu increase is maintained, and from this point of view, the results of the XPS and EDS analysis are in very good agreement. If the sample with the thinnest Cu layer is heat-treated (Cu 40 mA 50 s + T), the Cu concentration is decreased (from 17.6 at. % to 14.8 at. %). However, this decrease is relatively low and reveals that, if the wrinkle pattern is formed, a relatively large amount of Cu remains present on the biopolymer composite surface.

Surprisingly, the heat-treated PLLA sample sputtered with 100 s exhibited a lower Cu concentration than the sample deposited with half the time (14.8 at. % vs. 12.5 at. %). Although the decrease is not significant, one would expect the opposite dependence. It is also evident from Table 1 that, with an increasing amount of Cu on non-heated PLLA (sputtering time 300 s), there is a significant decrease in Cu after the heat treatment, which is caused by the diffusion of copper into the bulk wrinkles. Due to this phenomenon, the decrease is evident for XPS analysis data, when, for EDS data (obtained from a higher depth), no such significant effect was detected.

### 3.4. Wettability

**Figure 8:**
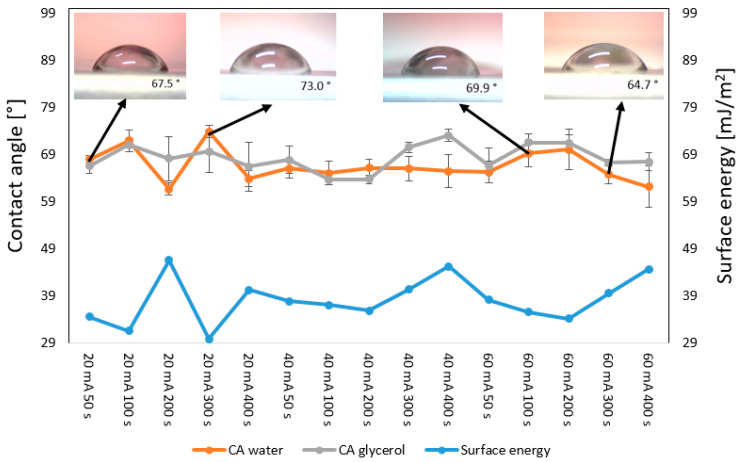
Wettability (contact angle and surface free energy) of sputtered PLLA with Cu and heat treated for 1 h and 60 °C: sputtering currents 20–60 mA with sputtering times from 50 s to 400 s; the samples were aged for 6 days. Representative drop photos are also displayed.

An important phenomenon with the ability to influence both surface antibacterial properties and cytocompatibility is the change in surface wettability, which can be determined by determining the contact angle and surface free energy. These changes are induced by surface morphology, surface chemistry, or a combination of both. It was previously determined by [49] that surface morphology can induce significant changes in wettability but only for surfaces with high roughness (micropatterns). This was not the case even for the samples sputtered with Cu with 60 mA and subsequently heat-treated; the surface wrinkle pattern did not increase in height by over one micron; therefore, we can suggest that the changes in wettability were induced mainly by the change in surface chemistry for all samples that were prepared on PLLA. As shown in Figure 8, the Cu-sputtered samples that were subsequently heat-treated, also after a six-day aging period, exhibited relatively stable surface values; the contact angle was 60–75°, and the surface free energy was 30–45 mJ·cm^−2^. This is important for any potential applications in tissue engineering, since these values are considered to be in an “optimal window” for successful cell adhesion and growth [50]. If there is no plasma pretreatment, but sputtering, e.g., with the combination of heat treatment, there are no significant variations in contact angle. We compared the contact angle values for samples that underwent aging, and there were fluctuations mostly within 5°; similar fluctuations occurred further in time, after 14 days. Since these fluctuations were significantly smaller compared with the possible fluctuations that can be achieved for plasma- or laser-treated samples, we chose to show those after 6 days. The data for wettability immediately after sputtering and heat treatment are introduced in Figure S3.

**Figure 9:**
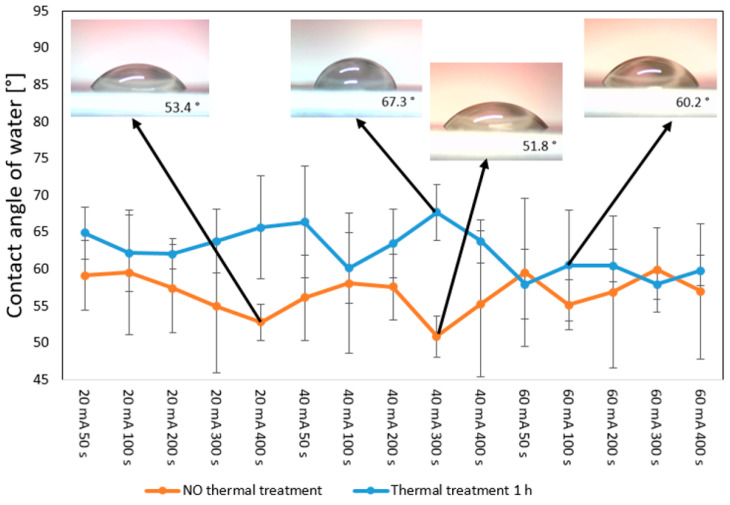
Wettability (contact angle) of sputtered PLLA with Cu: sputtering currents 20–60 mA with sputtering times from 50 s to 400 s (NO thermal treatment), also the samples were heat-treated at 60 °C for 1 h (Thermal treatment 1 h). Representative drop photos are also displayed.

Figure 9 shows the influence of heat treatment on changes in water contact angle for the Cu-sputtered samples with different Cu thicknesses and comparison with as-sputtered samples. From these data, it is evident that the surface chemistry (sputtered Cu and oxygen present in the layer) is mainly responsible for the changes in contact angle values. The contact angle for the Cu-PLLA surface was found to be between 50° and 60°. The values for the water contact angle of pure Cu were reported to be higher than these values [51]. Since the values for the water contact angle of the Cu layer were observed to be strongly dependent on the substrate itself, different values were determined, e.g., on glass/plasma-treated glass [52] and several other surfaces, as presented in [53]. The heat treatment of the Cu-PLLA system for most samples led to a slight increase in the contact angle from 60° to 70°. The samples deposited with the highest current 60 mA (and, therefore, a higher amount of Cu) exhibited similar values for the water contact angle before and after treatment. Even the wrinkle-like pattern is formed on the heat-treated PLLA: as revealed with EDS and XPS, the Cu is still present on the biopolymer surface, and only moderate changes in contact angle are, therefore, induced. Relevant information regarding surface wettability may be also acquired by determination of dynamic contact angle [54,55].

### 3.5. Antibacterial Properties

**Figure 10:**
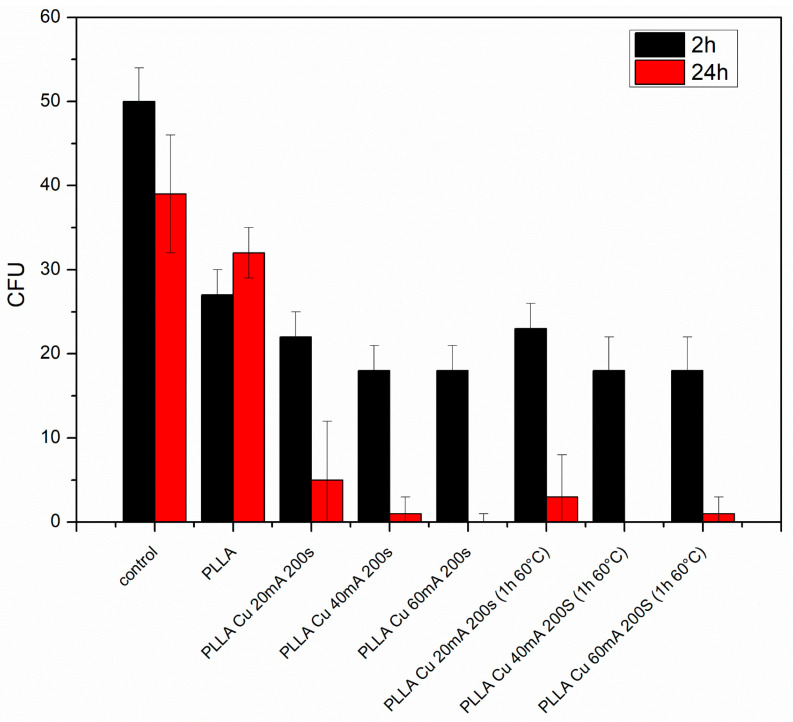
Antibacterial properties against Gram-negative *E. coli* for PLLA, sputtered PLLA samples (20 mA, 40 mA, and 60 mA for 200 s), and the same set of samples heat-treated for 1 h at 60 °C. Control sample is also shown; *y*-axis represents colony-forming units (CFUs).

**Figure 11:**
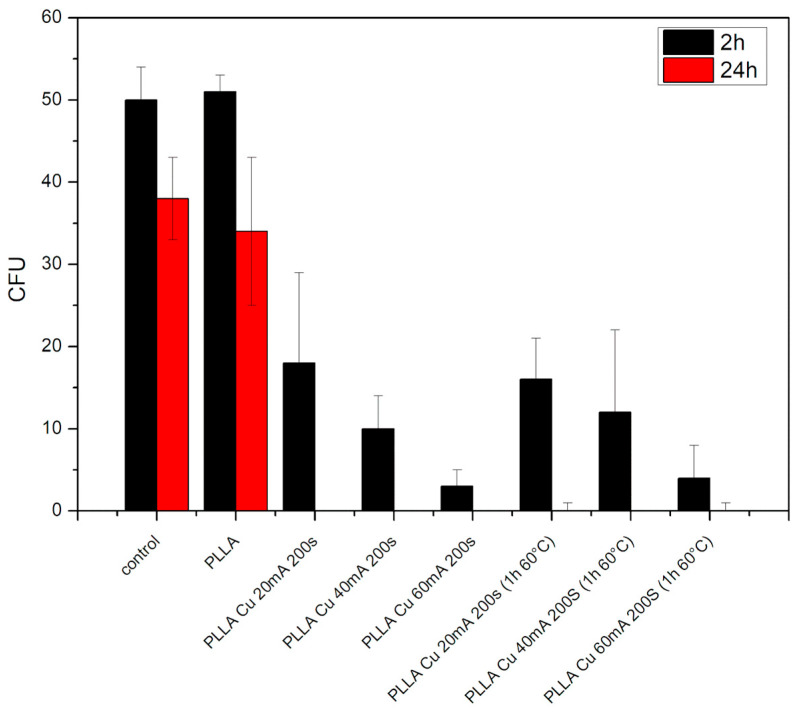
Antibacterial properties against G+ *S. aureus* for PLLA: sputtered PLLA samples (20 mA, 40 mA, and 60 mA for 200 s), and the same set of samples heat-treated for 1 h at 60 °C. Control sample is also displayed; *y*-axis represents colony-forming units (CFUs).

We focused our study on the antibacterial activity of prepared PLLA surface composites and simply sputtered surfaces against Gram-negative *E. coli* bacteria (Figure 10) and Gram-positive *S. aureus* bacteria (Figure 11). Our main goal at this stage was to prepare a biopolymer wrinkle pattern on a copper–poly-L-lactic basis as a strong antibacterial surface and to verify whether the patterning/wrinkling has an antibacterial effect or whether the simple sputter procedure alone is effective.

From this graph it is clear that simple copper sputtering on the biopolymer PLLA induces a significant antibacterial effect against *E. coli*. Moreover, the best antibacterial effect (complete diminishing of bacteria colonies) was observed for the sample with the highest Cu thickness (60 mA and 200 s) and the PLLA Cu 40 mA (+T) sample. However, after 240 h, almost all samples had a strong antibacterial effect against Gram-negative bacteria (Figure 10).

**Figure 12:**
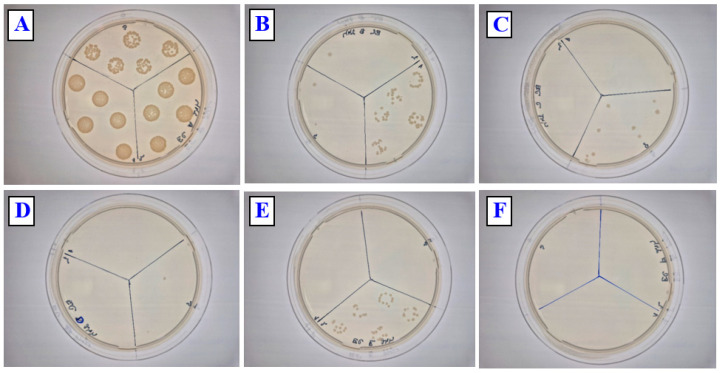
Representative photos of CFUs for PLLA (**A**), PLLA Cu 20 mA 200 s (**B**), PLLA Cu 40 mA 200 s (**C**), PLLA Cu 60 mA 200 s (**D**), PLLA Cu 20 mA 200 s (1 h 60 °C) (**E**), and PLLA Cu 40 mA 200 s (1 h 60 °C) (**F**), for *E. coli* strain after 24 h incubation. Numbers in mA represent the sputtering current, in combination with sputtering time in s.

**Figure 13:**
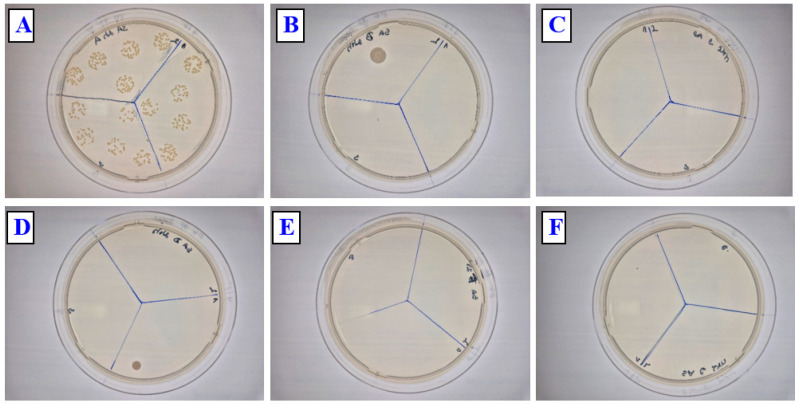
Representative photos of CFUs for PLLA (**A**), PLLA Cu 20 mA 200 s (**B**), PLLA Cu 40 mA 200 s (**C**), PLLA Cu 60 mA 200 s (**D**), PLLA Cu 20 mA 200 s (1 h 60 °C) (**E**), and PLLA Cu 40 mA 200 s (1 h 60 °C) (**F**) for *S. aureus* strain after 24 h incubation. Numbers in mA represent the sputtering current, in combination with sputtering time in s.

We also focused on the antibacterial activity of prepared PLLA surface composites against Gram-positive *S. aureus.* Simple copper sputtering on the biopolymer PLLA induces a significant antibacterial effect against *S. aureus* (Figure 11) compared with that against Gram-negative bacteria; this effect is more pronounced even after 2 h. After 24 h, the optimal antibacterial effect (complete diminishing of bacteria colonies) was observed for all samples with copper, both simply sputtered and/or combined with subsequent heat treatment. Representative photos of bacteria colonies are displayed in Figure 12 and Figure 13. It is evident that, for different thicknesses of Cu and for a combination with heat treatment, the optimal antibacterial effect prevails, so even when the Cu concentration on the surface is decreased due to the formation of a PLLA-Cu composite, the strong/ideal antibacterial effect prevails.

The antibacterial mechanism of nanostructured copper (Cu) has not been entirely elucidated, but current evidence suggests that it involves two complementary processes: (1) direct contact between bacterial cells and the metallic Cu surface, and (2) the release of Cu^+^/Cu^2+^ ions into the surrounding environment, followed by their interaction with microbial structures and biomolecules. The predominance of each mechanism depends on the physicochemical properties of the Cu surface and environmental conditions [56]. Surface morphology, roughness, and hydrophilic/hydrophobic character significantly influence the extent of the direct bactericidal effect. These surface parameters modulate interactions differently across Gram-positive and Gram-negative bacterial strains, largely due to structural differences in their cell walls. In particular, rough and hydrophilic surfaces enhance bacterial adhesion and subsequent disruption via direct metal contact [44]. Cu ions exert a broad-spectrum antibacterial action by targeting four critical components of the bacterial cell: the cell wall, cytoplasmic membrane, nucleic acids, and intracellular proteins. Cu ions can destabilize the peptidoglycan layer (especially in Gram-positive bacteria), disrupt membrane potential, and induce oxidative stress through the generation of reactive oxygen species (ROS). These effects collectively lead to membrane rupture and cell lysis [57]. This multi-target, multi-phase mechanism of copper’s antibacterial action contributes to its relatively low susceptibility to the development of bacterial resistance [58], making it a promising material for antimicrobial surface design and biomedical applications.

## 4. Conclusions

This study demonstrates the successful development of a novel antibacterial surface based on wrinkled copper–poly-L-lactic acid (Cu-PLLA) composites. The surface modification, achieved through copper sputtering followed by controlled thermal treatment at the glass transition temperature of PLLA, resulted in the formation of hierarchical wrinkle-like structures that enhance antibacterial efficacy. Comprehensive surface characterization using SEM, AFM, EDS, and XPS revealed a clear correlation between copper layer thickness, wrinkle morphology, and surface chemistry. Notably, the wrinkle formation was confirmed to be a result of bilayer instability, initiated by the thermal response of the Cu-PLLA interface and further governed by the thickness of the copper layer.

Despite the lack of significant surface roughness variation before heat treatment, the introduction of thermal wrinkling significantly increased the effective surface area and morphological heterogeneity. XPS analysis confirmed that, while some copper diffuses deeper into the polymer matrix during wrinkling, a substantial concentration remains on the surface, maintaining bactericidal activity. Importantly, the antibacterial assays conducted against both *Escherichia coli* and *Staphylococcus aureus* demonstrated that the synergy between copper’s inherent antimicrobial properties and the topographical cues provided by the wrinkle patterns leads to superior bactericidal performance.

Among the tested conditions, the surfaces with thicker copper layers and those subjected to thermal treatment showed the most significant reduction in bacterial viability. In particular, the complete suppression of bacterial colonies on samples with higher Cu content (e.g., 60 mA, 200 s) and heat-treated wrinkled surfaces underscores the critical role of engineered surface architecture in enhancing antimicrobial efficiency. Moreover, the observation that even thinner Cu-coated surfaces provided long-lasting antibacterial effects after 24 h highlights the stability and practical potential of these hybrid surfaces.

In conclusion, the formation of wrinkle patterns on Cu-PLLA surfaces not only provides a mechanical means to disrupt bacterial membranes but also ensures sustained exposure to copper ions, creating a dual-mode antibacterial mechanism. These findings position Cu-PLLA wrinkled composites as promising candidates for use in biomedical applications, including as implant coatings, wound dressings, and antibacterial packaging materials. Future work may focus on long-term stability and biocompatibility studies and on extending this approach to other biopolymer/metal systems for broader applicability.

## Figures and Tables

**Table 1:** Surface elemental chemistry (XPS spectra) of sputtered pristine PLLA and PLLA sputtered with Cu with particular combinations of sputtering current, sputtering time, and heat treatment. Atomic concentrations are introduced.

	C [at. %]	O [at. %]	Cu [at. %]
Pristine PLLA	62.5	37.5	
PLLA + Cu 40 mA 50 s	40.6	41.8	17.6
PLLA + Cu 40 mA 50 s + T	49.7	35.5	14.8
PLLA + Cu 40 mA 300 s	38.4	35.7	26
PLLA + Cu 40 mA 100 s + T	50.6	36.9	12.5
PLLA + Cu 40 mA 300 s + T	50.9	34	15.1

## Data Availability

The data are available at https://doi.org/10.5281/zenodo.15872583.

## Supplementary Materials

The following supporting information can be downloaded at: https://www.mdpi.com/article/10.3390/polym17162173/s1, Figure S1. Surface morphology of sputtered PLLA with Cu and subsequently heat treated (1 h, 60 °C): sputtering current 40 mA (50 s and 300 s). The effective surface area (S), average surface roughness (Ra), and root mean square roughness (RMS) are shown; Figure S2. The surface elemental chemistry (XPS spectra) of sputtered pristine PLLA, PLLA sputtered with Cu, the sputtering current 40 mA with sputtering time 50 s and 300 s, and the sample sputtered with 40 mA for 50 s and subsequently heat treated at 60 °C for 1 h (T); Figure S3. Wettability (contact angle) of sputtered PLLA with Cu and heat treated for 1 h and 60 °C: sputtering currents 20–60 mA with sputtering times from 50 s to 400 s; the samples were measured after deposition (day 1) and aged for 6 days.
