# Supplementary material for: Copper–PLLA-Based Biopolymer Wrinkle Structures for Enhanced Antibacterial Activity
Source: Polymers (Basel). 2025 Aug 8;17(16):2173. doi: 10.3390/polym17162173 (PMC12389648; doi:10.3390/polym17162173)
Supplement: Supplementary file 1 [file polymers-17-02173-s001.zip › polymers-3756478-supplementary.pdf]

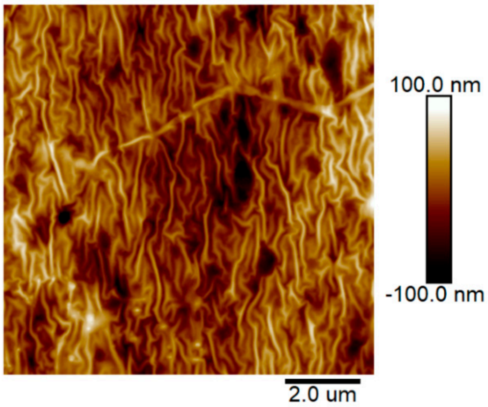	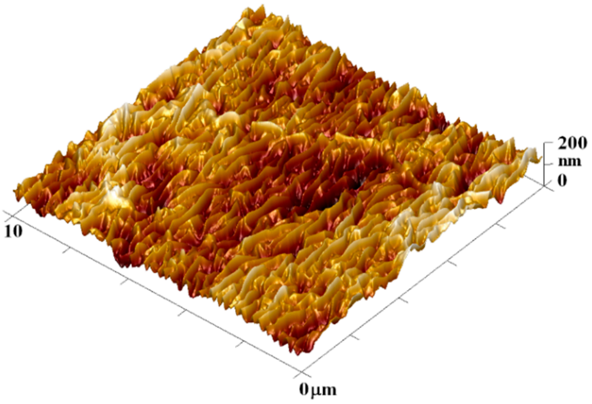
PLLA_Cu_40mA_50s_1h60C 10 µm 2D<br>S = 106 µm^2 (5.90 %)	PLLA_Cu_40mA_50s_1h60C 10 µm 3D<br>R_a = 20.8 nm; RMS = 26.4 nm
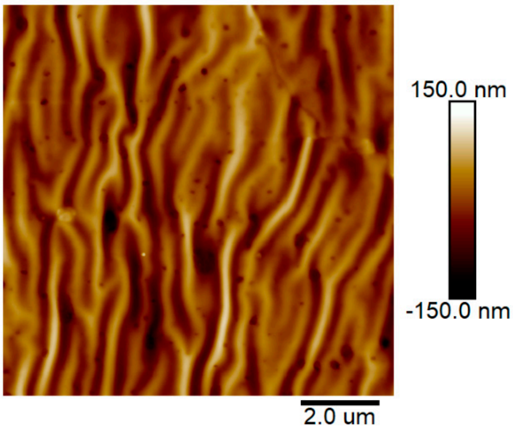	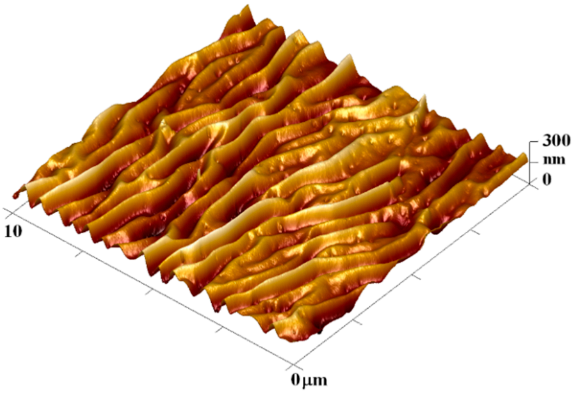
PLLA_Cu_40mA_300s_1h60C 10 µm 2D<br>S = 105 µm^2 (4.49 %)	PLLA_Cu_40mA_300s_1h60C 10 µm 3D<br>R_a = 27.2 nm; RMS = 33.7 nm

Figure S1. Surface morphology of sputtered PLLA with Cu and subsequently heat treated (1 hour, 60° C): sputtering current 40 mA (50 s and 300 s). The effective surface area (S), average surface roughness (R_a), and root mean square roughness (RMS) are shown.

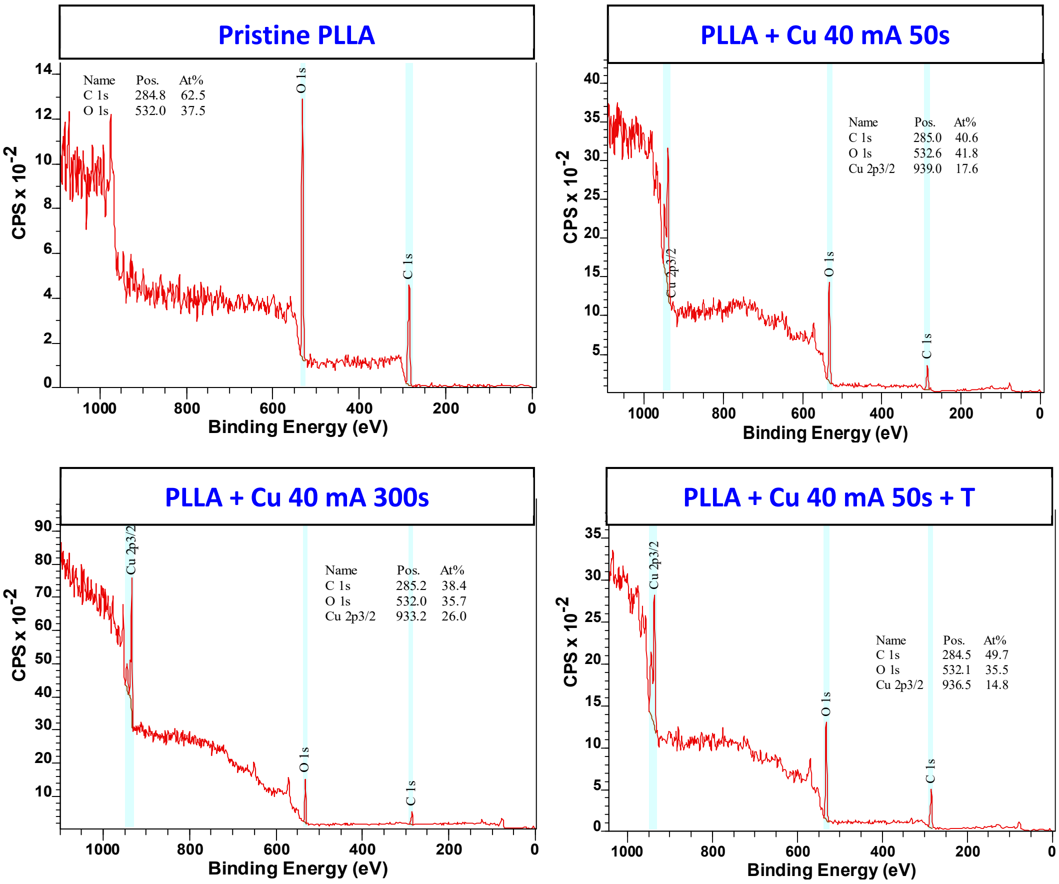

Figure S2. The surface elemental chemistry (XPS spectra) of sputtered pristine PLLA, PLLA sputtered with Cu, the sputtering current 40 mA with sputtering time 50 s and 300 s, and the sample sputtered with 40 mA for 50 s and subsequently heat treated at 60 °C for 1 hour (T).

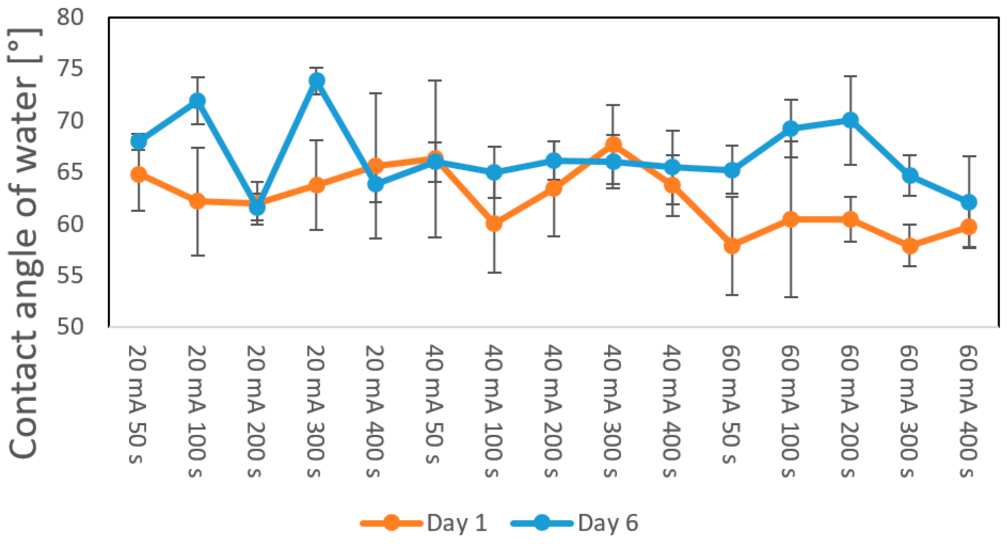

Figure S3. Wettability (contact angle) of sputtered PLLA with Cu and heat treated for 1 h and 60°C: sputtering currents 20–60 mA with sputtering times from 50 s to 400 s; the samples were measured after deposition (day 1) and aged for 6 days.
